# Pulmonary Cryptococcosis Diagnosed by Metagenomic Next-Generation Sequencing in a Young Patient With Normal Immune Function: A Case Report

**DOI:** 10.3389/fpubh.2022.942282

**Published:** 2022-07-22

**Authors:** Yingyu Zhang, Weiliang Wang, Yingxuan Zhang, Sina Zhai, Han Xia, Xilin Zhang

**Affiliations:** ^1^Department of Tuberculosis, Foshan Fourth People's Hospital, Foshan, China; ^2^Department of Scientific Affairs, Hugobiotech, Beijing, China

**Keywords:** cryptococcosis, *Cryptococcus neoformans*, immune competent patients, metagenomic next-generation sequencing, diagnosis

## Abstract

**Background:**

Pulmonary cryptococcosis (PC) is a serious opportunistic fungal infection that usually occurs in immunocompromised patients. This disease is often difficult to diagnose in time due to its clinical manifestations and radiological feature similar to other pulmonary infections, as well as the low sensitivity of conventional diagnostic methods. Cryptococcosis in immune-competent patients is rare.

**Case Presentation:**

Here we report a case of PC in an immune-competent patient. Tuberculosis was suspected according to radiological features due to the positive T-lymphocyte spot test and pure protein derivative skin test. To further detect the pathogen, bronchoalveolar lavage fluid (BALF) was collected for metagenomic next-generation sequencing (mNGS). *Cryptococcus neoformans* (one specific read) was identified by mNGS, indicating the PC of this patient. The following BALF culture and cryptococcal antigen lateral flow assay (CrAg-LFA) test also showed *Cryptococcus* infection, confirming the mNGS detection. Voriconazole (0.4 g daily) was given orally according to the subsequent susceptibility results. After seven months of treatment, the patient's condition improved.

**Conclusion:**

Metagenomic next-generation sequencing (mNGS) is a better diagnostic tool to help clinicians distinguish pulmonary cryptococcosis from other atypical pulmonary infections.

## Background

Pulmonary cryptococcosis (PC) is a subacute or chronic pulmonary fungal infection mainly caused by *Cryptococcus neoformans* (1, 2). This disease appears more likely to occur in immunocompromised patients, such as patients with human immunodeficiency virus (HIV) infection (3). There are one million new cases of cryptococcosis per year worldwide, and the mortality rate reaches 20–70% (3, 4). The clinical manifestations and imaging changes of PC usually lack specificity and are difficult to be distinguished from bacterial pneumonia, pulmonary tuberculosis, and lung tumors, resulting in a delay in diagnosis or misdiagnosis (2, 5–7). Early and accurate diagnosis is the key to improving the cure rate and prognosis. Conventional methods, such as culture, are commonly used for the diagnosis of PC, but they often have poor timeliness and low sensitivity.

Here, we report a 16-year-old immune-competent male patient with PC. The mNGS was used and detected the pathogen as *C. neoformans*, indicating PC. The result was then verified by bronchoalveolar lavage fluid (BALF) culture and cryptococcal antigen lateral flow assay (CrAg-LFA). Finally, according to the minimum inhibitory concentration (MIC) index of *C. neoformans* culture results, voriconazole was given for antifungal treatment.

## Case Presentation

**Figure 1:**
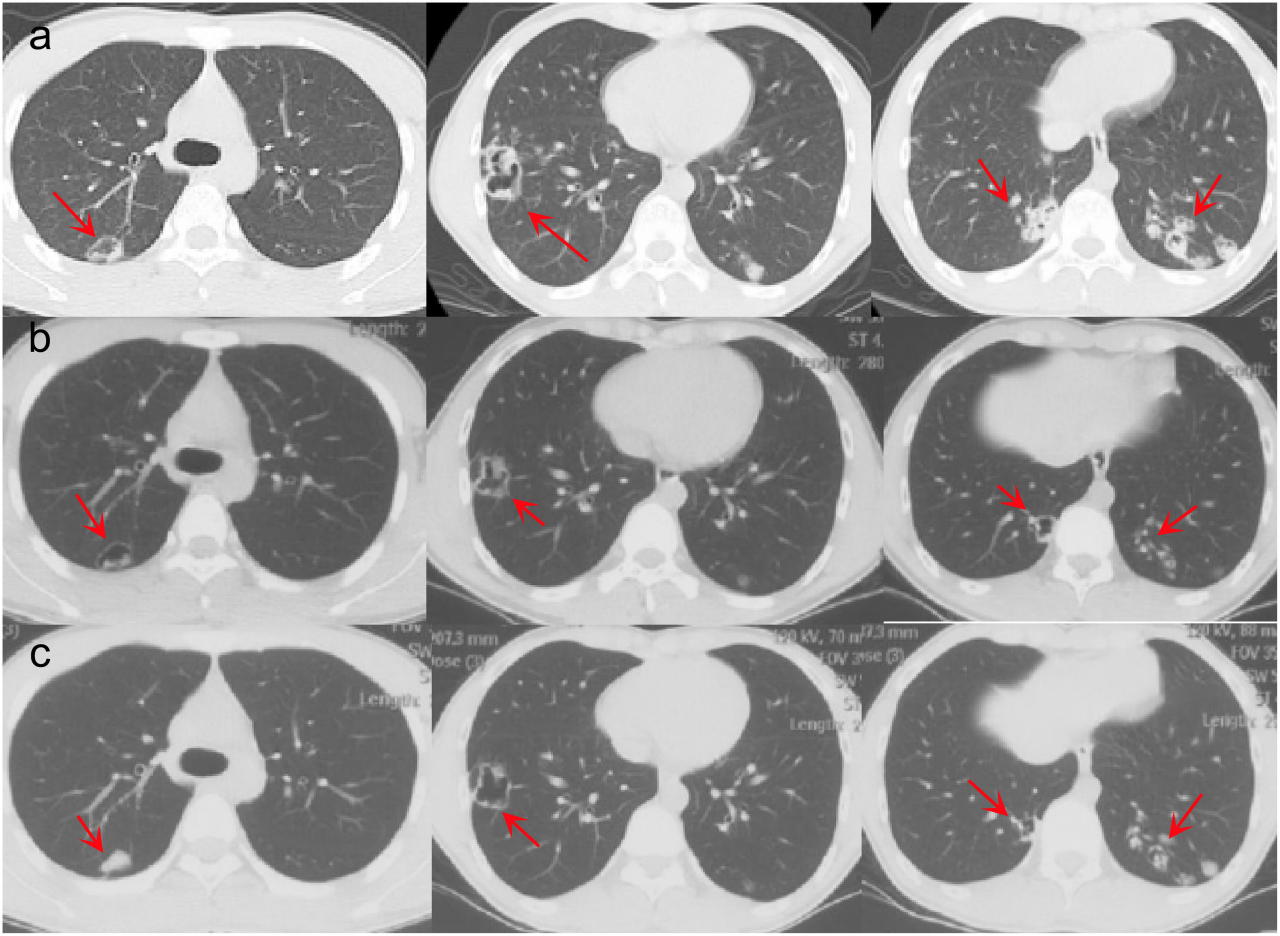
Pre- and post-therapy CT scan. **(a)** Chest CT before treatment. **(b)** Chest CT after cefotaxime sodium sulbactam treatment. **(c)** Chest CT after voriconazole treatment.

**Figure 2:**
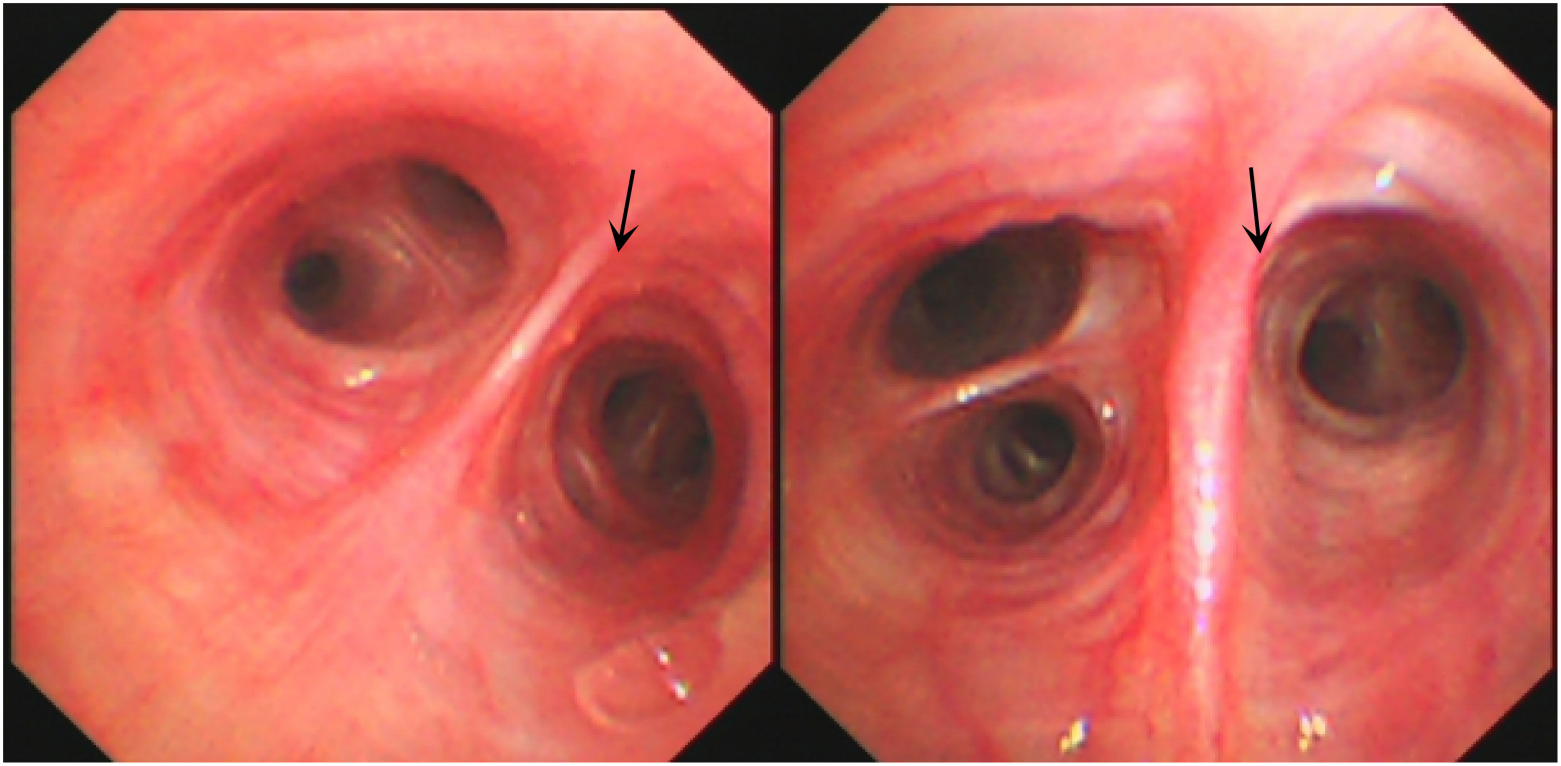
Bronchoscopic image on November 17th **(left)** and 20th **(right)**, 2020.

A 16-year-old male student from Jieyang City, Guangdong Province was admitted to Foshan Fourth People's Hospital on 11 November 2020. The patient had an intermittent cough without obvious inducement 6 months ago, mainly dry cough, no fever, chest pain, or other adverse symptoms. He was previously healthy, with no history of bird feces or soil contact. On 19 October 2020, the chest CT examination was conducted at the local hospital, which revealed chest shadow and suspected tuberculosis. Then, he came to our hospital for treatment. There were no obvious abnormalities on physical examination after admission. The blood assay showed a white cell count of 7.20 × 10^9^/L, neutrophils of 51.9% (3.73 × 10^9^/L), and C-reactive protein of 9 mg/L. T-lymphocyte spot test (T-SPOT) and pure protein derivative (PPD) skin test (13 × 14 mm) were positive. In terms of bacteriology, sputum acid-fast staining was negative on 13, 14, and 15 November 2020. On 13 November 2020, a chest plain scan and enhanced CT showed left lower lobe and right pulmonary cavities lesions (Figure 1a), which suggested possible pyogenic inflammation, fungal infection, or secondary tuberculosis. The patient was given the diagnostic anti-infective treatment of cefotaxime sodium sulbactam 2.25 g twice a day. After 10 days of regular treatment, the chest CT scan showed little change compared with pre-treatment (Figure 1b). There were no significant abnormalities in the basic immunosuppressive status assessment (immunoglobulin level, T lymphocyte subsets, HIV detection, etc.). On 17 November 2020, a bronchoscopic examination showed that the bronchial mucosa of each lobe and subsegment on both sides was congestive and edematous (Figure 2). Microscopic examination of bronchial lavage fluid (BALF) showed a medium amount of squamous epithelium, bronchial columnar epithelial cells, and a small number of neutrophils, but no cancer cells. Microscopic examination of tracheoscopic brush film revealed a medium amount of columnar epithelial cells and no cancer cells. These results excluded etiologies other than infection.

The etiological examination using BALF revealed that GeneXpert (-): MTB-DNA: negative, rpoB: negative. G test revealed <37.50 pg/ml. No acid-fast bacilli, bacteria, spores, or hyphae were found. Examination and diagnosis: bronchitis changes.

**Figure 3:**
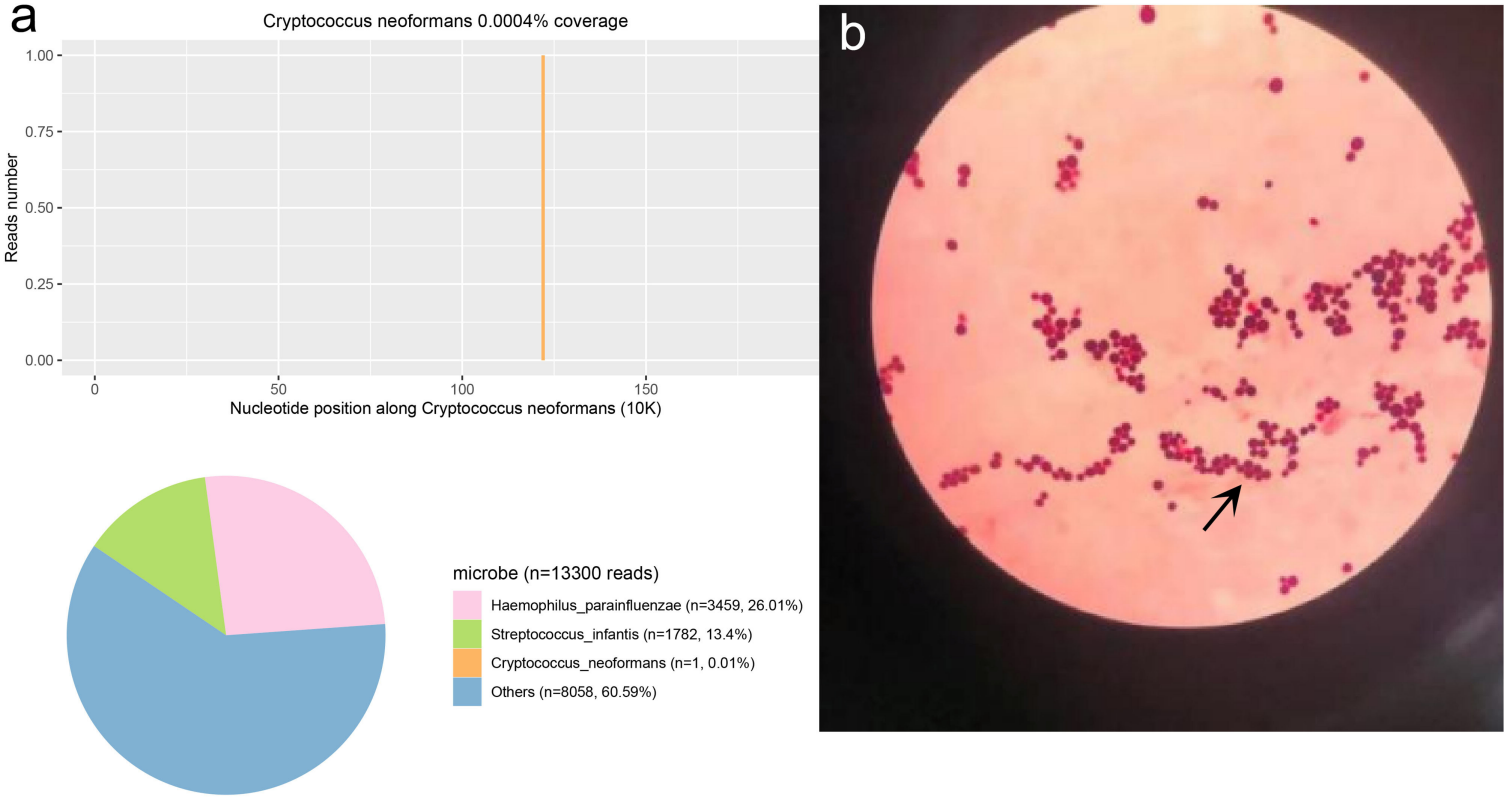
The *Cryptococcus neoformans* mNGS and culture result. **(a)** The mNGS result of *C. neoformans*. 0.01% of bacterial reads corresponded to *C. neoformans* with a coverage of 0.0004%. **(b)** The results of the fungi smear showed *C. neoformans*.

**Figure 4:**
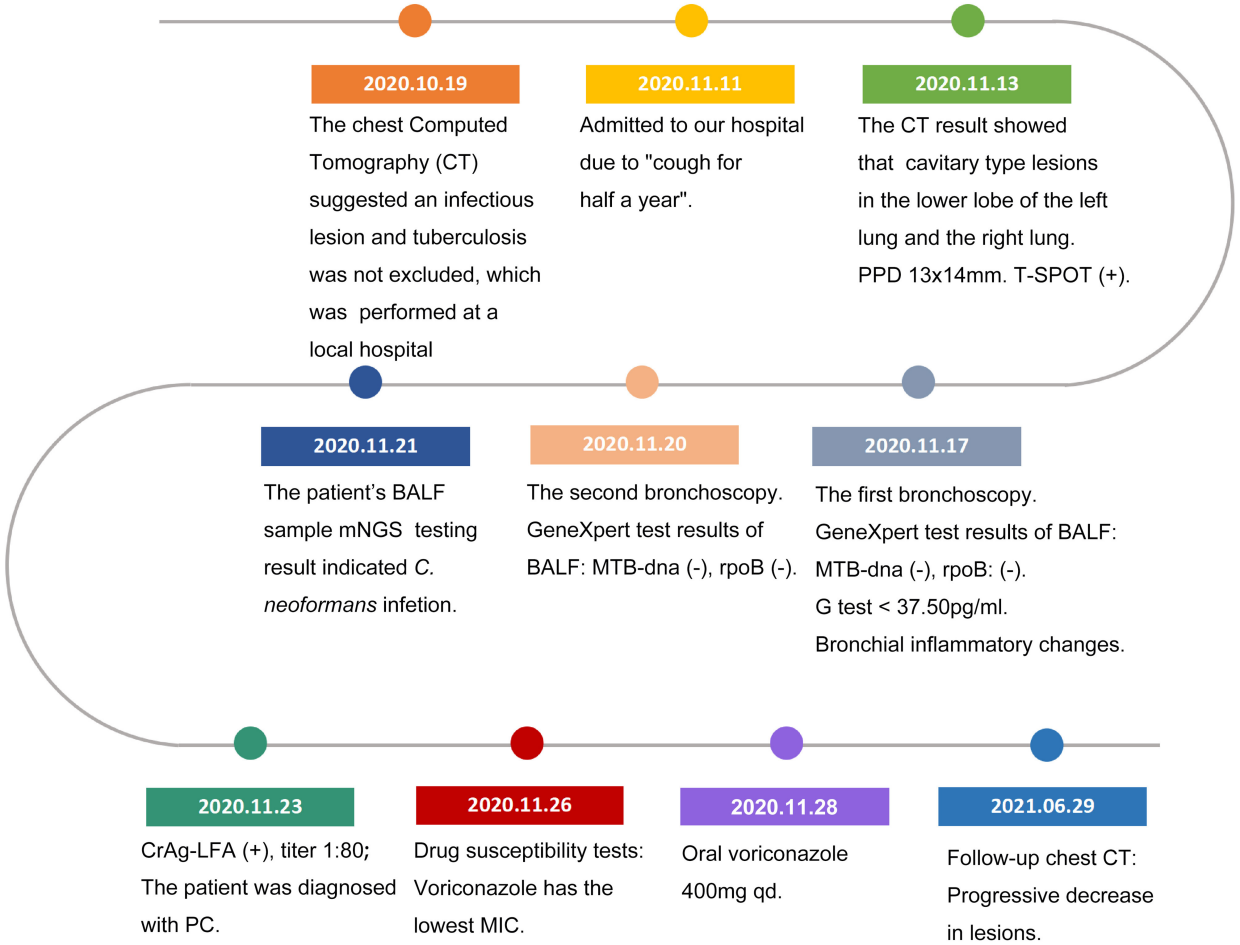
Timeline of the reported case.

The second tracheoscopy on 20 November 2020, indicated bilateral bronchitis. BALF GeneXpert was negative. The BALF was then sent for PACEseq mNGS (Hugobiotech, Beijing, China) on a Nextseq 550 platform (Illumina). On 21 November 2020, the mNGS result came back and showed one specific read of *C. neoformans* (Figure 3a). On 23 November 2020, CrAg-LFA (titer 1:80) and fungal culture (creamy mucoid colonies with relatively smooth edges on Sabouraud Dextrose Agar medium, initially colorless and which appeared lavender with time after staining using CHRMagaar color plate) revealed as positive, confirming the mNGS detection (Figure 3b). The patient was diagnosed with PC. The drug susceptibility results on 26 November 2020, suggested that this strain was sensitive to voriconazole (MIC ≤ 0.13), itraconazole (MIC < 0.25), fluconazole (MIC ≤ 4), and amphotericin B (MIC < 0.5), among which voriconazole had the lowest MIC (Table 1). Therefore, the patient was given oral voriconazole of 400 mg QD on 28 November 2020 for seven months. On 29 June 2021, a chest CT was performed and showed a progressive decrease in lesions of the patient (Figure 1c). The whole treatment process was shown in Figure 4.

**Table 1:** Drug susceptibility results of *Cryptococcus neoformans*.

**Antibacterial agents**	**MIC**	**Result**
Itraconazole	<0.25	Sensitive
Voriconazole	≤ 0.13	Sensitive
Amphotericin B	<0.5	Sensitive
5-Fluorocytosine	<4	Sensitive
Fluconazole	≤ 4	Sensitive

## Discussion and Conclusions

*C. neoformans* is widely distributed in soil and bird feces and can cause lung infection by inhalation of spores (8). Although progressively detected in immunocompetent patients, the majority of *C. neoformans* infections occur in immunocompromised patients. PC is still under-diagnosed due to limitations in diagnostic tools and similar characteristics to lung neoplasms and TB radiologically (2). PC with pulmonary cavities is common in patients with hypoimmunity, but rare in non-immunosuppressive patients (9). In this case, the patient was not immunosuppressive and had no history of exposure to soil or bird feces. So, PC was not first considered. Conventional diagnostic methods for infectious or non-infectious causes failed to identify the etiology. mNGS was then used and indicated *C. neoformans* infection, which was confirmed by CrAg-LFA (+) and fungal culture (+). After a drug susceptibility test, the treatment was adjusted to voriconazole, and the symptoms were relieved.

The efficiency of conventional diagnostic methods is not satisfactory, and the high misdiagnosis rate of PC in China has been described (10). Lung biopsy is the gold standard for the diagnosis of pulmonary cryptococcosis, but the patients will suffer from a lot of pain and have the risk of complications from puncture. Currently, CrAg-LFA has been widely applied for the diagnosis of cryptococcosis with relatively high sensitivity and specificity (11). However, it needs a prior hypothesis of the pathogen, which is difficult in this case. The mNGS was first applied in the clinical diagnosis of infections in 2014 (12). This approach is culture-independent and can rapidly detect almost all known microbes (including bacteria, viruses, fungi, and parasites) in one run (13). The mNGS displays significant advantages in detecting various infections, including PC (14–16). In this study, BALF-mNGS successfully detected the pathogen as *C. neoformans*, which was confirmed by fungal culture and CrAg-LFA test. Lung biopsy has been avoided.

There were some limitations to this case report. The etiology of PC in this patient is unclear. The patient is a high school student with normal immune function and lives in a simple and clean environment. He declared no history of exposure to soil and bird feces. The source of *C. neoformans* infection remains unknown. CT revealed right pulmonary cavities lesions in this case, which are common in patients with tuberculosis, lung cancer, lung abscess, and mycosis (17). PC with pulmonary cavities in patients with normal immune function is rare. More samples are needed to explore the etiology of PC in patients with normal immune function. Besides, mNGS was not first considered in this case. The main reason is that the cost of mNGS is significantly higher than that of conventional diagnostic methods. However, it shortened the time of diagnosis in this patient, so that a guided accurate medication reduced the risk of disease progression. The length of hospital stay has also been shortened, reducing the economic burden on the patient. Thus, mNGS should be applied as quickly as possible, especially for patients with severe infections for an unknown reason.

Voriconazole, itraconazole, and amphotericin B are commonly recommended for the treatment of cryptococcal diseases (18). In this case, the antibiotic susceptibility test also suggested that the strain was most sensitive to voriconazole. After 7 months of treatment using voriconazole, the conditions of the patient improved. Regular follow-up continued due to his extensive lesions.

In conclusion, this case has led to a new contemplation of immune-competent PC patients with vague clinical symptoms. mNGS may be a potential diagnostic method for patients with PC.

## Data Availability Statement

The datasets presented in this study can be found in online repositories. The names of the repository/repositories and accession number(s) can be found at: https://ngdc.cncb.ac.cn/, PRJCA009558.

## Ethics Statement

This study was approved by the Ethical Review Committee of Foshan Fourth People's Hospital. Written informed consent to participate in this study was provided by the participants' legal guardian/next of kin.

## Author Contributions

YingyZ and XZ designed and drafted the paper. YingyZ, WW, YingxZ, SZ, and XZ are involved in the clinical care and management of the patient. HX analyzed the mNGS data. All authors approved the final manuscript as submitted and agree to be accountable for all aspects of the work.

## Conflict of Interest

HX was employed by Hugobiotech Co., Ltd. The remaining authors declare that the research was conducted in the absence of any commercial or financial relationships that could be construed as a potential conflict of interest.

## Publisher's Note

All claims expressed in this article are solely those of the authors and do not necessarily represent those of their affiliated organizations, or those of the publisher, the editors and the reviewers. Any product that may be evaluated in this article, or claim that may be made by its manufacturer, is not guaranteed or endorsed by the publisher.
